# A combination of aerobic and resistance training induces white adipose tissue browning

**DOI:** 10.1007/s13105-026-01234-7

**Published:** 2026-09-17

**Authors:** Caroline de Carvalho Picoli, Gustavo Renan Gilio, Felipe Henriques, Kaltinaitis B N H Santos, Magno Alves Lopes, Solange Marta Franzói de Moraes, Luzmarina Hernandes, Miguel Luiz Batista, Sidney Barnabé Peres

**Affiliations:** 1 Center for Molecular Medicine, Maine Health Institute for Research, Scarborough, ME USA; 2 Institute of Biomedical Sciences, University of São Paulo, São Paulo, Brazil; 3 Program in Molecular Medicine, University of Massachusetts Medical School, Worcester, MA USA; 4 Department of Integrated Biotechnology Group, University of Mogi das Cruzes, Mogi das Cruzes, São Paulo Brazil; 5 Department of Physiological Sciences, State University of Maringá, Maringá, Paraná Brazil; 6 Department of Morphological Sciences, State University of Maringá, Maringá, Paraná Brazil; 7 Bonds Biosystems, Strathmore Rd, Natick, MA USA

**Keywords:** UCP1, Beige adipocytes, Exercise, Combined training

## Abstract

Combined training (CT) has been widely recommended as a strategy to improve metabolic health and is often prescribed in the context of obesity and its comorbidities. The present study aimed to evaluate the effects of CT on the induction of brown-like adipocytes and white adipose tissue (WAT) remodeling. Sixty male Swiss mice were randomly assigned to two experimental groups: sedentary (SED; *n* = 30) and combined training (CT; *n* = 30). Animals underwent incremental and load tests at the beginning of each week to adjust training intensity. The total CT training period lasted 8 weeks, with sessions performed five days a week. After euthanasia, WAT from the inguinal and retroperitoneal (iWAT and rpWAT) were harvested. Performance in the CT group was improved after 8 weeks, as indicated by an increase in V_peak_k_ and the load lifted. The expression of vascular endothelial growth factor (VEGF), cluster of differentiation 31 (CD31), and uncoupling protein 1 (UCP1) was higher in the CT group than in the SED group. CT also increased the abundance of multilocular adipocytes and the expression of genes associated with brown and beige adipocyte phenotypes in iWAT and rpWAT. Together, these findings indicate that CT induces browning in iWAT and rpWAT in lean mice, supporting its potential relevance as a strategy for promoting WAT remodeling in the context of metabolic health.

## Key Points

Combined training (CT) integrates aerobic and resistance exercise and is an effective strategy to improve physical performance.

CT increased the expression of angiogenesis-associated markers (VEGF and CD31) in white adipose tissue (WAT).

CT induced the appearance of multilocular adipocytes and upregulated uncoupling protein 1 (UCP1) along with brown and beige phenotype-specific genes in both inguinal (iWAT) and retroperitoneal (rpWAT) depots.

CT drives WAT remodeling and browning across multiple fat depots, supporting its potential relevance to metabolic health, which remains to be directly tested.

## Introduction

Aerobic exercise training is widely recognized for enhancing cardiovascular health and respiratory efficiency [60], and improving glucose metabolism [50, 52, 57, 61]. Moreover, it is also an efficient strategy for reducing body weight and is particularly effective in reducing body fat [17, 30, 42, 73, 75]. On the other hand, resistance exercise training is promotes several muscular adaptations, such as preserving muscle mass, enhancing muscular strength, muscle fiber hypertrophy, and increasing bone density [48, 63].

Although aerobic exercise training has been most often used to reduce body weight and induce the remodeling of white adipose tissue (WAT), resistance exercise training has also been shown to be effective in promoting weight loss [44, 54]. The American College of Sports Medicine recommends the combination of both types of training to enhance weight loss outcomes and improve metabolic health [29]. Therefore, strategies to combat obesity and co-morbidities have also suggested this type of prescription as an effective approach [10, 36].

Combined training (CT), which involves both aerobic and resistance exercise, can be performed within the same training program or period. When these exercise modalities are performed on the same day or session, the protocol is often referred to as concurrent training [16]. This approach may lead to either additive or competing physiological adaptations, depending on how the aerobic and resistance stimuli interact to create an ‘interference effect’ influencing the maximal strength gains [9, 11, 28, 43].

Previous studies evaluating CT on adipose tissue outcomes have predominantly used same-day protocols across different animal models and adipose tissue depots, with heterogeneous results [1, 10, 26, 53]. To our knowledge, few studies have evaluated an alternate-day, weekly periodized CT protocol in relation to WAT browning in healthy animals. We have previously shown that both aerobic and resistance training performed separately can promote the browning of inguinal and retroperitoneal (iWAT and rpWAT) depots, in a similar phenotype [59]. In the present study, we employed a forced and periodized CT protocol, on alternate days within the same week, rather than in the same session, with the aim of reducing the close temporal overlap between aerobic and resistance stimuli. However, whether CT prescribed in the same period (week), using alternate days for aerobic and resistance exercise, in a forced and periodized manner, could induce significant browning of WAT in lean rodents remains unknown.

## Materials and methods

### Ethical approval

**Fig. 1:**
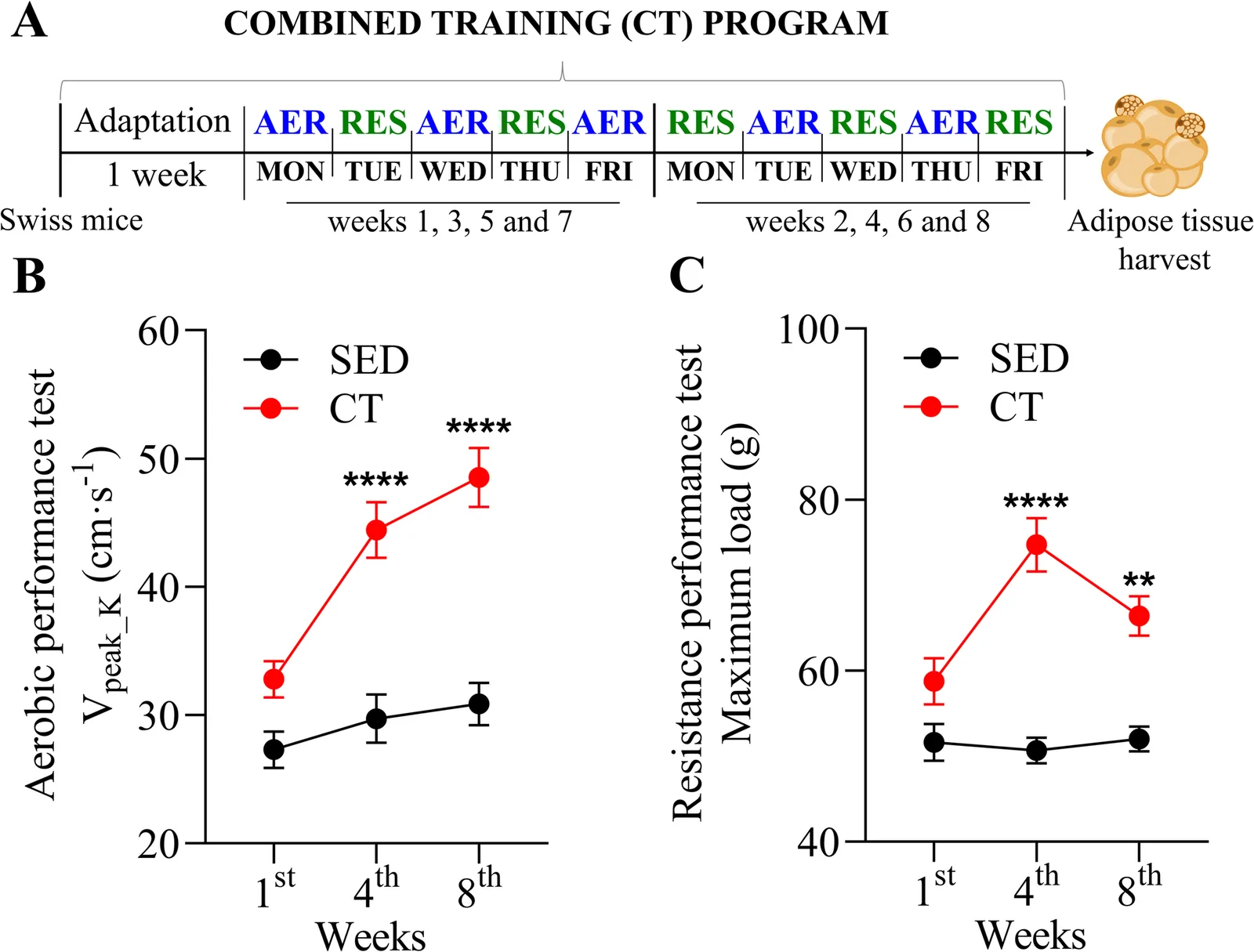
Improvement in exercise training performance after 8 weeks of CT exercise. V_peak_K_ and maximum load lifted were determined during the 1^st^, 4^th^, and 8^th^ weeks. (**A**) Combined training protocol and experimental design, (**B**) Aerobic performance was assessed by peak velocity during the incremental test (V_peak_K_); (**C**) Resistance performance was assessed by the maximum load lifted (g). Data are presented as mean ± SD and were analyzed using two-way repeated-measures ANOVA, followed by Šídák’s multiple-comparisons test. **p < 0.01 and ****p < 0.0001 versus the corresponding SED group at the same time point. Abbreviations: SED, sedentary; CT, combined training; AER, aerobic training; RES, resistance training. SED, n = 10 animals; CT, n = 10 animals

All experimental procedures were approved by the Ethics Committee of the State University of Maringa (protocol nº 033/2014). Figure 1 A shows graphical representations of the experimental designs. Furthermore, all experimental procedures were conducted in compliance with the regulations of the National Council for the Control of Animal Experimentation (CONCEA-Brazil) and followed the ARRIVE 2.0 Guidelines for reporting in vivo animal research [56].

### Animals and experimental design

**Fig. 2:**
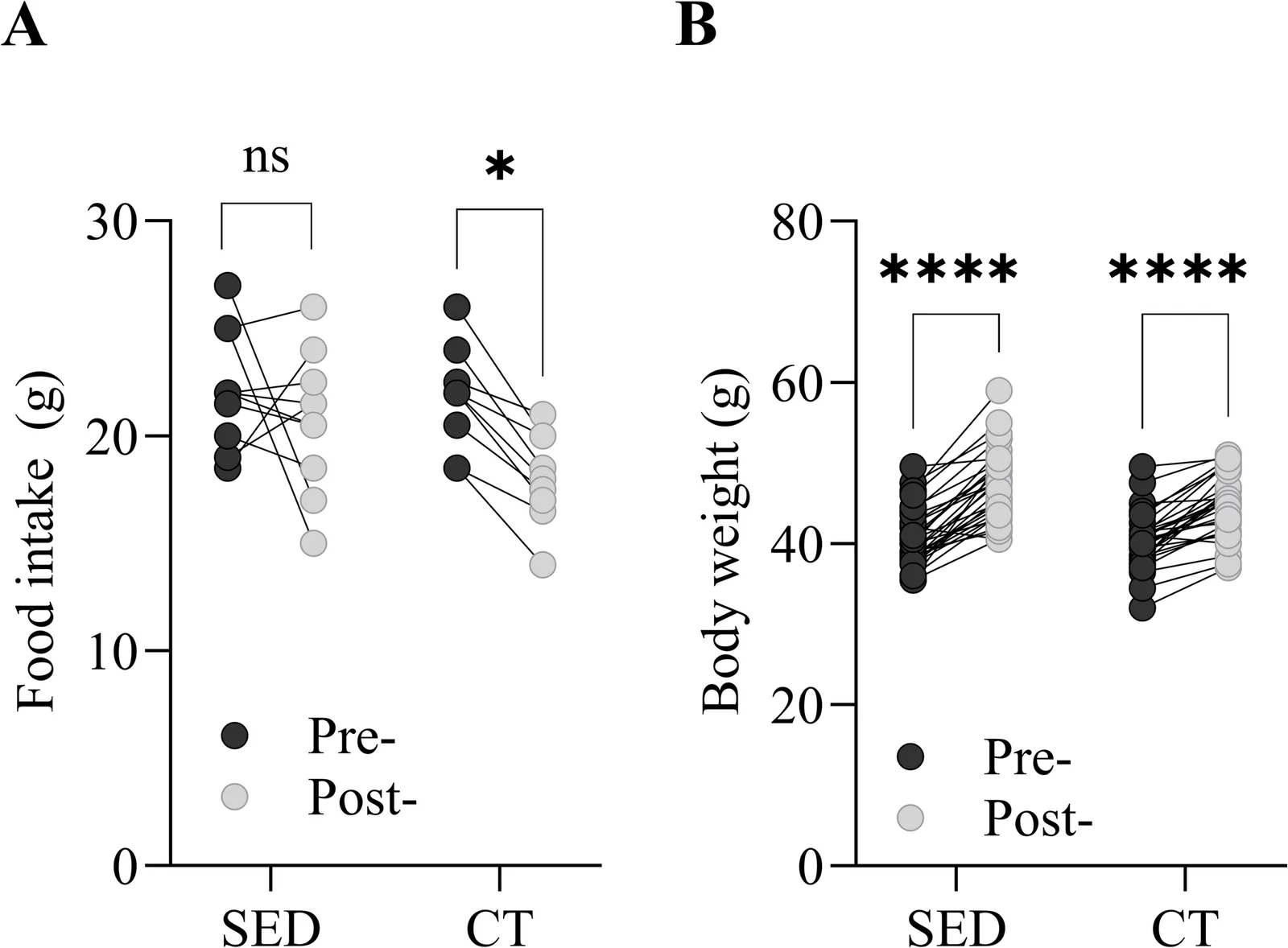
Food intake and body mass before and after 8 weeks of CT. (**A**) Food intake was assessed in SED (n = 10) and CT (n = 10) animals. (**B**) Body mass was assessed in SED (n = 30) and CT (n = 30) animals. Data are presented as mean ± SD and were analyzed using two-way repeated-measures ANOVA, followed by Šídák’s multiple-comparisons test. *p < 0.05 and ****p < 0.0001 versus the respective pre-training value. Abbreviations: SED, sedentary; CT, combined training; Pre, pre-training; Post, post-training

Sixty 45-day-old male Swiss mice were randomly assigned to sedentary (SED; *n* = 30) and combined training (CT; *n* = 30). All mice were maintained in individual polypropylene cages, with bedding cleaned weekly, in an automated room for photoperiod control (light-dark cycle 12/12-h) at 21–23 °C. The mice were allowed to feed (Nuvilab Cr1^®^) and drink water *ad libitum.* Before the initiation of performance testing or the training intervention, 10 animals from each experimental group were randomly selected for longitudinal performance comparison and food-intake assessments. The same animals were evaluated at all time points. Body mass was assessed in all 30 animals per group throughout the experimental protocol, whereas the food-intake analysis presented in Fig. 2A was based on this predefined subset of 10 animals per group.

### Combined training protocol

For longitudinal performance comparisons, the same predefined subset of 10 animals per group was used throughout the study. CT was performed alternately between aerobic and resistance training, 5 days per week, in all trained mice. In weeks 1, 3, 5, and 7, aerobic training was performed on Monday, Wednesday, and Friday, and resistance training on Tuesday and Thursday. In weeks 2, 4, 6, and 8, this sequence was reversed (Fig. 1A). CT was performed once daily, starting at 6 p.m. during the dark cycle, for a total of 40 training sessions.

Aerobic training was prescribed as previously described [58]. Mice from both the SED and CT groups underwent treadmill familiarization for 3 consecutive days, followed by incremental testing to determine peak velocity (V_peak_K_). These tests were performed in both groups at the corresponding assessment time points to allow longitudinal comparison of aerobic performance. In the CT group, the incremental test was additionally used to adjust training intensity. Only the CT group subsequently underwent aerobic training at 70% of V_peak_K_. Sessions lasted 30–40 min in week 1, 35–50 min in week 2, 50–60 min in week 3, and 60 min from week 4 through week 8, with a 0° slope.

For resistance training, mice from both the SED and CT groups underwent 3 consecutive days of adaptation to ladder climbing, followed by an incremental test to determine the maximum load lifted. These procedures were performed in both groups to allow longitudinal comparison of resistance performance. Only the CT group subsequently underwent resistance training. Training consisted of 8–12 dynamic movements performed in four sets at 50%, 75%, 90%, and 100% of the maximum load lifted, with training loads adjusted according to the results of the incremental tests [37].

### Experimental procedures

At the end of the experimental period, the animals were weighed and anesthetized with a lethal mixture of ketamine (80 mg/kg body mass) and xylazine (15 mg/kg body mass) administered intraperitoneally. Afterward, animals underwent a median laparotomy for the removal of inguinal and retroperitoneal (iWAT and rpWAT) depots.

### Adipocyte area quantification

At the end of the experimental period, inguinal and retroperitoneal (iWAT and rpWAT) depots were weighed and fixed in 4% paraformaldehyde (pH 7.4) for 3 h and processed for inclusion in paraffin and semi-serial cuts of 5 μm. Adipocyte area was quantified from hematoxylin and eosin (H&E)-stained sections of iWAT and rpWAT. Images were acquired using a light microscope at a fixed magnification across all samples. For each animal, multiple non-overlapping fields were randomly selected from comparable anatomical regions to ensure representative sampling. Adipocyte area was measured using ImageJ software (Version 1.56, NIH, United States). A minimum number of 100 adipocytes per animal was analyzed to ensure robust estimation of mean adipocyte size. The mean adipocyte area for each animal was used for statistical analysis. For immunohistochemical and immunofluorescence analyses, six animals per group were initially allocated. Following tissue processing and quality control, one animal per group was excluded. This exclusion was consistent across all techniques. Therefore, the final sample size was five animals per group for immunohistochemistry and immunofluorescence.

### Immunohistochemistry and immunofluorescence

The harvested inguinal and retroperitoneal (iWAT and rpWAT) depots were fixed in 4% paraformaldehyde (pH 7.4) for 3 h and processed for inclusion in paraffin and semi-serial cuts of 5 μm. Subsequently, samples were deparaffinized and hydrated to block peroxidase. The following primary antibodies (Abcam, Cambridge, UK) were used for immunohistochemistry: anti-VEGF (dilution 1:300/ab1316) and anti-UCP1 (dilution 1:300/ab10983). The commercial Histostain^®^ Plus kit (Histostain Plus Mouse Primary DAB Zymed) was used to reveal staining, according to the manufacturer’s protocol, and sections were counterstained with Mayer’s hematoxylin (Sigma). The sections were analyzed with a Nikon Eclipse E110 microscope. The following primary antibodies were used for immunofluorescence: CD31-PE (dilution 1:100/no. 558736), purchased from BD Biosciences, and DAPI (dilution 1:1,000/28718-90-3), purchased from Life Technologies, and mounted in Dako fluorescence mounting medium; confocal images were acquired on a Zeiss 880. The immunoreactivity was quantified using Image-Pro Plus software (Media Cybernetics, Maryland, USA) by identifying positive-staining pixels within each analyzed field. The positive pixels were converted to area and expressed as positive-staining area per field (µm²).

### Quantitative real-time PCR

The total RNA of inguinal and retroperitoneal (iWAT and rpWAT) depots was isolated using the PureLink^®^ RNA Mini Kit (Invitrogen^®^ Life Technologies) following the procedure described by the manufacturer. For gene expression analyses, adipose tissue samples from two mice belonging to the same experimental group were combined to generate one pooled biological sample. After six mice per group were allocated to histological analyses, tissue from the remaining 24 animals per group was used to generate 12 pooled biological samples per group. iWAT and rpWAT were processed separately. Following RNA extraction, DNase treatment, and quality control, some pooled samples did not meet the RNA quantity or quality criteria required for downstream analysis. In addition, individual qPCR reactions were excluded when they showed unacceptable amplification or melting-curve profiles consistent with nonspecific amplification. Therefore, the final number of pooled biological samples varied according to the target gene, experimental group, and adipose tissue depot. The exact sample size for each analysis is reported in the Fig. 6 legend. The quantification of RNA in the samples was performed by a spectrophotometric reading of the absorbance at 260 nm, and the samples were stored at −80 °C. Reverse transcription (RT) was performed using the High-Capacity cDNA Reverse Transcription kit (Invitrogen^®^ Life Technologies) following the procedure described by the manufacturer. All incubations were performed on Eppendorf thermal cycler, and the material (cDNA) was kept at −20 °C. The quantification of the mRNA of genes of interest was done by the real-time PCR methodology using the Power SYBR^®^ Green PCR Master Mix kit (Invitrogen^®^ Life Technologies), the ABI PRISM 7700 Sequence Detection System (Applied Biosystems, Foster City, CA), and specific primer pairs (direct and reverse) for the target genes. *Ribosomal protein L19 (RPL19)* was used as a housekeeping gene for normalization, based on previous studies demonstrating stable expression in adipose tissue under metabolic and exercise-related conditions [3, 32]. The relative expression of the data was calculated by the delta-delta Ct method (2-ΔΔCT algorithm) [47].

The following primers for the mouse target genes were used: *Ucp1* sense: GGGACGTCATCTGCCAGT and antisense: GAAAGGCAAATGACCCCAAG; *Pgc1α* sense: AGCCGTGACCACTGACAACGAG and antisense: GCTGCATGGTTCTGAGTGCTAAG; *Prdm16* sense: CAGCACGGTGAAGCCATTC and antisense: GCGTGCATCCGCTTGTG; *Cidea* sense: TGCTCTTCTGTATCGCCCAGT and antisense: GCCGTGTTAAGGAATCTGCTG; *Eva1* sense: GTCCCAACCAGACCATCAAC and antisense: CTCCA CTTGCTCTGGAAGC; *Cd137/Tnfrs9* sense: CGTGCAGAACTCCTGTGATAAC and antisense: GTCCACCTATGCTGGAGAAGG; *Tmem26* sense: ACCCTGTCATCCCACAGAG and antisense: TGTTTGGTGGAGTCCTAAGGTC; *Tbx1* sense: GGCAGGCAGACGAATGTTC and antisense: TTGTCATCTACGGGCACAAAG; *RPL19* sense: CTGATCAAGGATGGGCTGAT and antisense: ACCCTTCCTCTTCCCTATGC.

### Statistical analysis

Sample size was calculated a priori using G*Power software (v.3.1.9.7) [23], based on a two-tailed independent-samples t-test for UCP1 immunohistochemistry. The anticipated effect size was estimated from prior UCP1 data, yielding a Cohen’s d of 2.24. Assuming an allocation ratio of 1:1, an α level of 0.05, and 80% power, the calculation indicated a minimum sample size of 5 animals per group. Data normality was assessed using the Shapiro–Wilk test. Comparisons between the SED and CT groups for outcomes assessed at a single time point were performed using an unpaired two-tailed Student’s t-test. Non-normally distributed outcomes assessed at a single time point were analyzed using the Mann–Whitney U test. Outcomes measured in the same animals over time were analyzed using two-way repeated-measures ANOVA, with group as the between-subject factor and time as the within-subject factor, followed by Šídák’s multiple-comparisons test when appropriate [68].

For the gene-expression analyses presented in Fig. 6, Welch’s unpaired t-tests were performed for each molecular marker [76]. Multiple testing across the eight markers was controlled separately within each adipose tissue depot using the two-stage step-up false discovery rate (FDR) procedure of Benjamini, Krieger, and Yekutieli, with Q set at 5% [7]. Data are presented as mean ± standard deviation (SD). Statistical significance was set at *p* < 0.05 for individual analyses, whereas FDR-adjusted significance for the gene expression panel was defined according to Q = 5%. All statistical analyses were performed using the GraphPad Prism 8.0 software (GraphPad Software, San Diego, CA).

## Results

### Training performance, food intake, and body mass

CT improved aerobic and resistance performance over time, with significant training × time interactions for V_peak_K_ (*p* = 0.0058) and maximum load lifted (*p* = 0.0150). Multiple comparisons showed greater performance in the CT group than in the SED group in weeks 4 and 8 (Figs. 1B and C).

Food intake showed a significant main effect of time (*p* = 0.0034), whereas neither the main effect of training group (*p* = 0.2291) nor the time × training group interaction (*p* = 0.5374) was significant. Multiple comparisons showed a significant reduction from pre- to post-training in the CT group (*p* < 0.001), whereas no significant change was observed in the SED group (Fig. 2A). However, the nonsignificant interaction indicates that the magnitude of change did not differ significantly between groups. At the end of the experimental protocol, body weight showed a significant main effect of time (*p* < 0.0001) and a significant time × training group interaction (*p* = 0.0078), whereas the main effect of training group was not significant (*p* = 0.1086). Multiple comparisons showed a significant increase in body weight from pre- to post-training in both the SED and CT groups (*p* < 0.0001). No significant differences were observed between the groups at the pre- or post-training time points (Fig. 2B). The increase in body weight could reflect the normal growth of the mice during the experimental period [22].

### WAT Morphology, immunohistochemistry and immunofluorescence

CT reduced adipocyte size in iWAT without affecting depot mass. In rpWAT, CT significantly decreased both depot mass and adipocyte area compared with the SED group (*p* < 0.05) (Table 1).

**Table 1:** Fat pad mass and adipocyte area of WAT

		SED	CT
iWAT	mass (g)	0.362 ± 0.02	0.319 ± 0.02
	area (µm^2^)	2252.0 ± 469.1	666.7 ± 63.16*
rpWAT	mass (g)	0.242 ± 0.02	0.176 ± 0.01*
	area (µm^2^)	2028.0 ± 244.0	1101.0 ± 50.54*

Abbreviation: *SED* sedentary (*n* = 5 animals), *CT* Combined training exercise (*n* = 5 animals), *iWAT* inguinal white adipose tissue, *rpWAT* retroperitoneal white adipose tissue. Values are presented as mean ± SD. *p < 0.05; significant difference between groups (t-test)

**Fig. 3:**
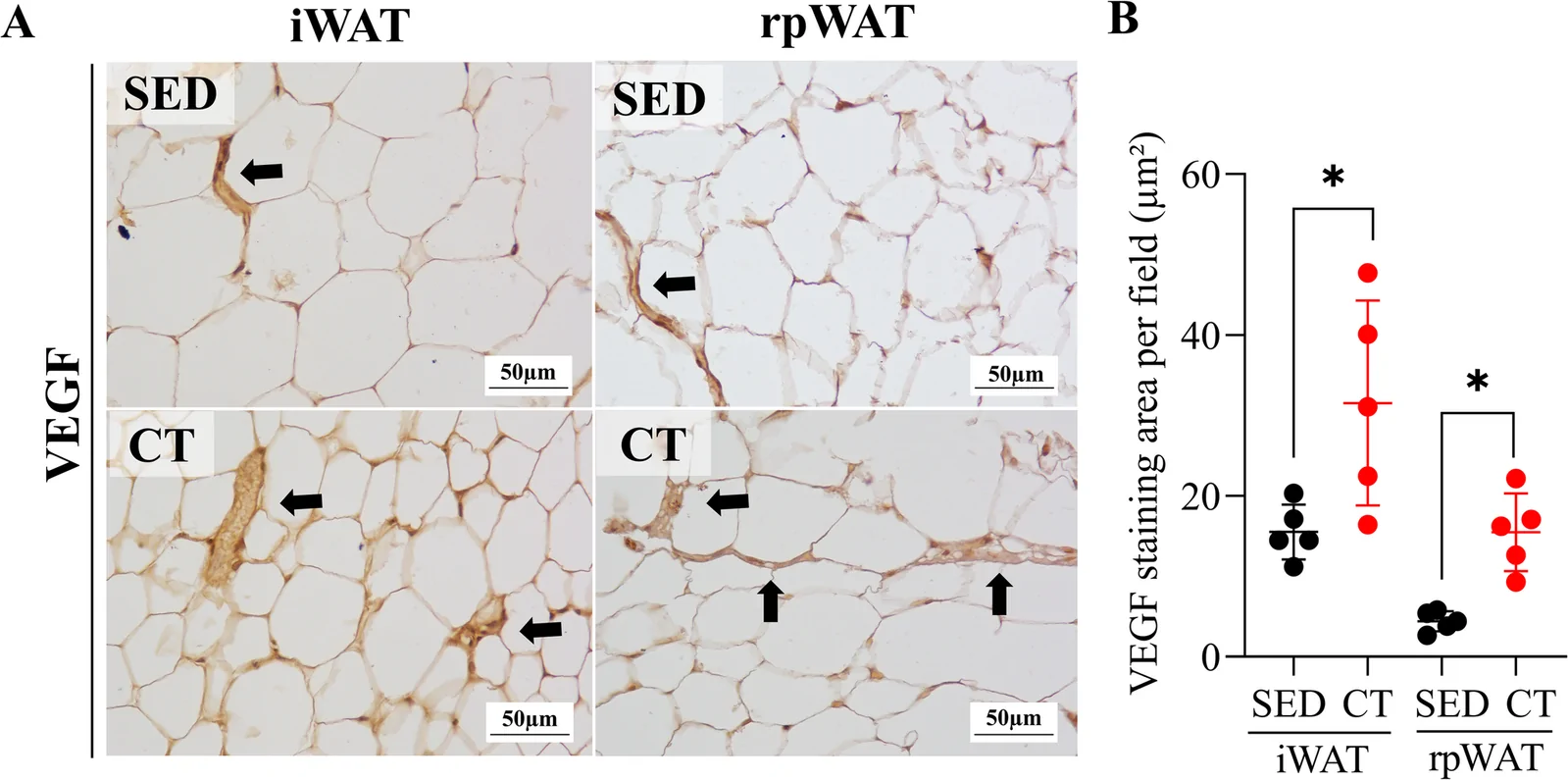
CT increased VEGF expression in WAT depots after 8 weeks. (**A**) VEGF was detected by immunohistochemistry; (**B**) VEGF positive-staining area per field (µm²) after 8 weeks of CT. Data are presented as mean ± SD. Comparisons between SED and CT were performed using an unpaired two-tailed Student’s t-test. *p < 0.05 versus the corresponding SED group. The reported sample size corresponds to the final number of animals with successfully processed and analyzed tissue sections. Black arrows indicate VEGF-positive structures. Scale bars, 50 μm. Abbreviations: SED, sedentary (n = 5 animals); CT, combined training (n = 5 animals); WAT, white adipose tissue; iWAT, inguinal white adipose tissue; rpWAT, retroperitoneal white adipose tissue

**Fig. 4:**
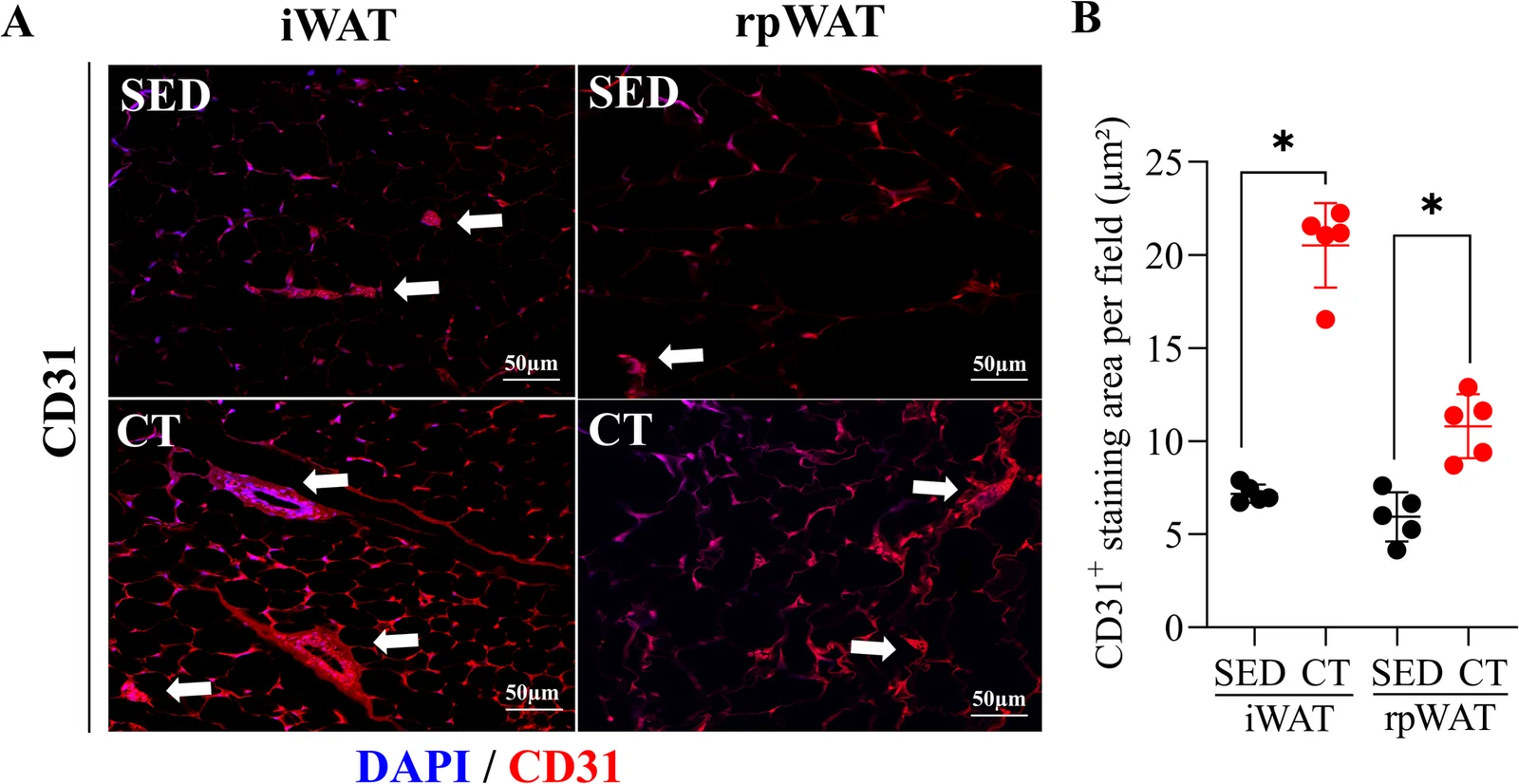
CT enhances CD31 expression in WAT after 8 weeks. (**A**) Representative immunofluorescence images of CD31 staining in iWAT and rpWAT; (**B**) CD31 positive-staining area per field (µm²) in each adipose tissue depot. Data are presented as mean ± SD. Comparisons between SED and CT were performed using an unpaired two-tailed Student’s t-test. *p < 0.05 versus the corresponding SED group. The reported sample size corresponds to the final number of animals with successfully processed and analyzed tissue sections. White arrows indicate CD31-positive structures. Scale bars, 50 μm. Abbreviations: SED, sedentary (n = 5 animals); CT, combined training (n = 5 animals); WAT, white adipose tissue; iWAT, inguinal white adipose tissue; rpWAT, retroperitoneal white adipose tissue; DAPI, 4′,6-diamidino-2-phenylindole; CD31, cluster of differentiation 31

**Fig. 5:**
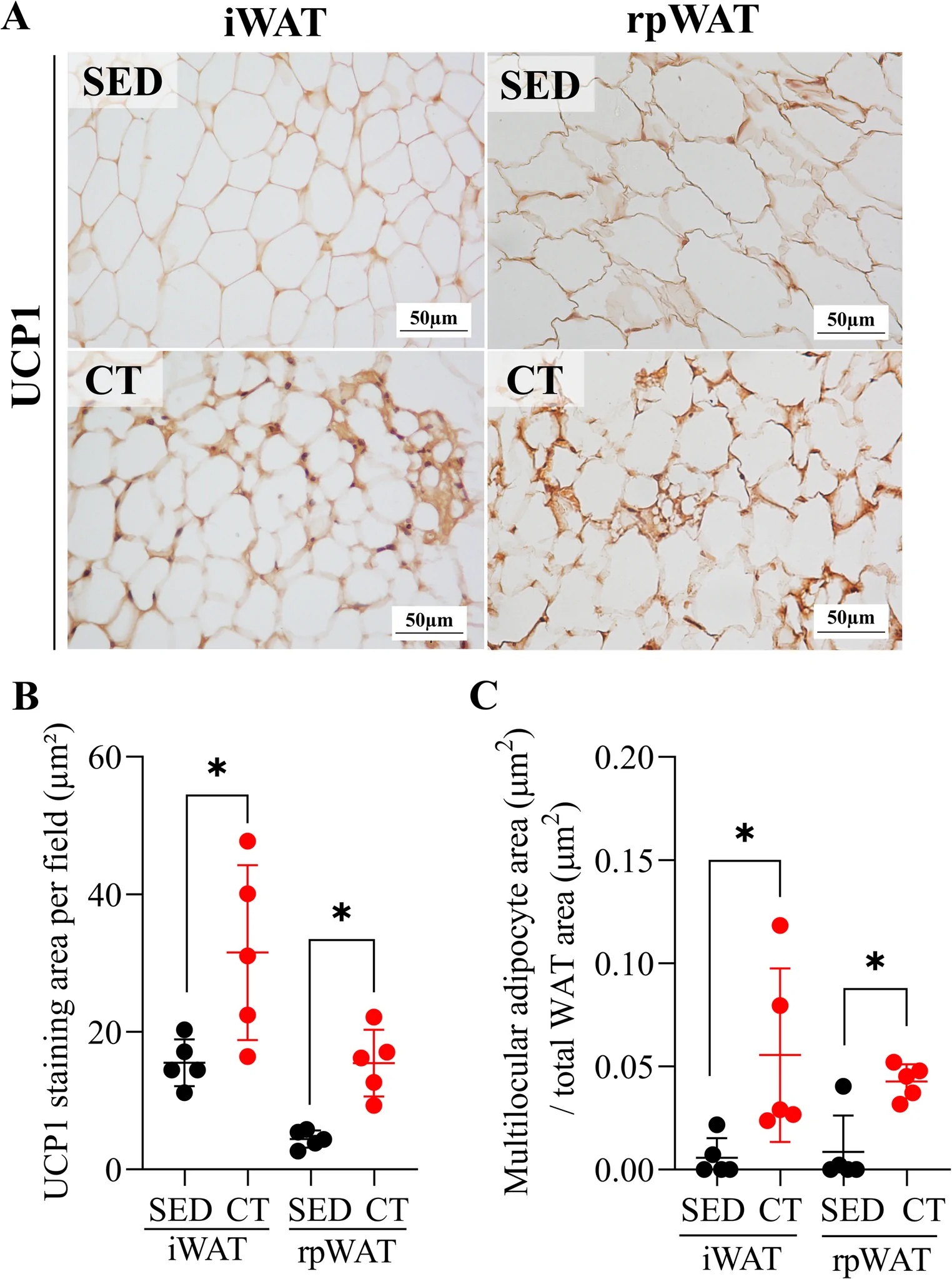
CT increased UCP1 expression in iWAT and rpWAT after 8 weeks. (**A**) Representative immunohistochemistry images of UCP1 staining in iWAT and rpWAT; (**B**) UCP1 positive-staining area per field (µm²) in each adipose tissue depot; and (**C**) Multilocular adipocyte area normalized to the total WAT area analyzed. Data are presented as mean ± SD. Comparisons between SED and CT were performed using an unpaired two-tailed Student’s t-test. *p < 0.05 versus the corresponding SED group. The reported sample size corresponds to the final number of animals with successfully processed and analyzed tissue sections. Scale bars, 50 μm. Abbreviations: SED, sedentary (n = 5 animals); CT, combined training (n = 5 animals); WAT, white adipose tissue; iWAT, inguinal white adipose tissue; rpWAT, retroperitoneal white adipose tissue; UCP1, uncoupling protein 1

The browning phenotype of WAT is associated with angiogenesis or blood vessel formation [71]. The vascular endothelial growth factor (VEGF) and the cluster of differentiation 31 (CD31) are bona fide markers of angiogenesis. Both markers were enhanced in iWAT and rpWAT after 8 weeks of CT, consistent with changes in angiogenesis-associated markers (*p* < 0.05) (Figs. 3 and 4). In parallel, UCP1 expression was significantly elevated, and multilocular adipocyte abundance was increased in iWAT and rpWAT following CT (*p* < 0.05), consistent with a browning phenotype (Fig. 5).

### Expression of selective genes related to brown phenotype and beige cell lineage origin

**Fig. 6:**
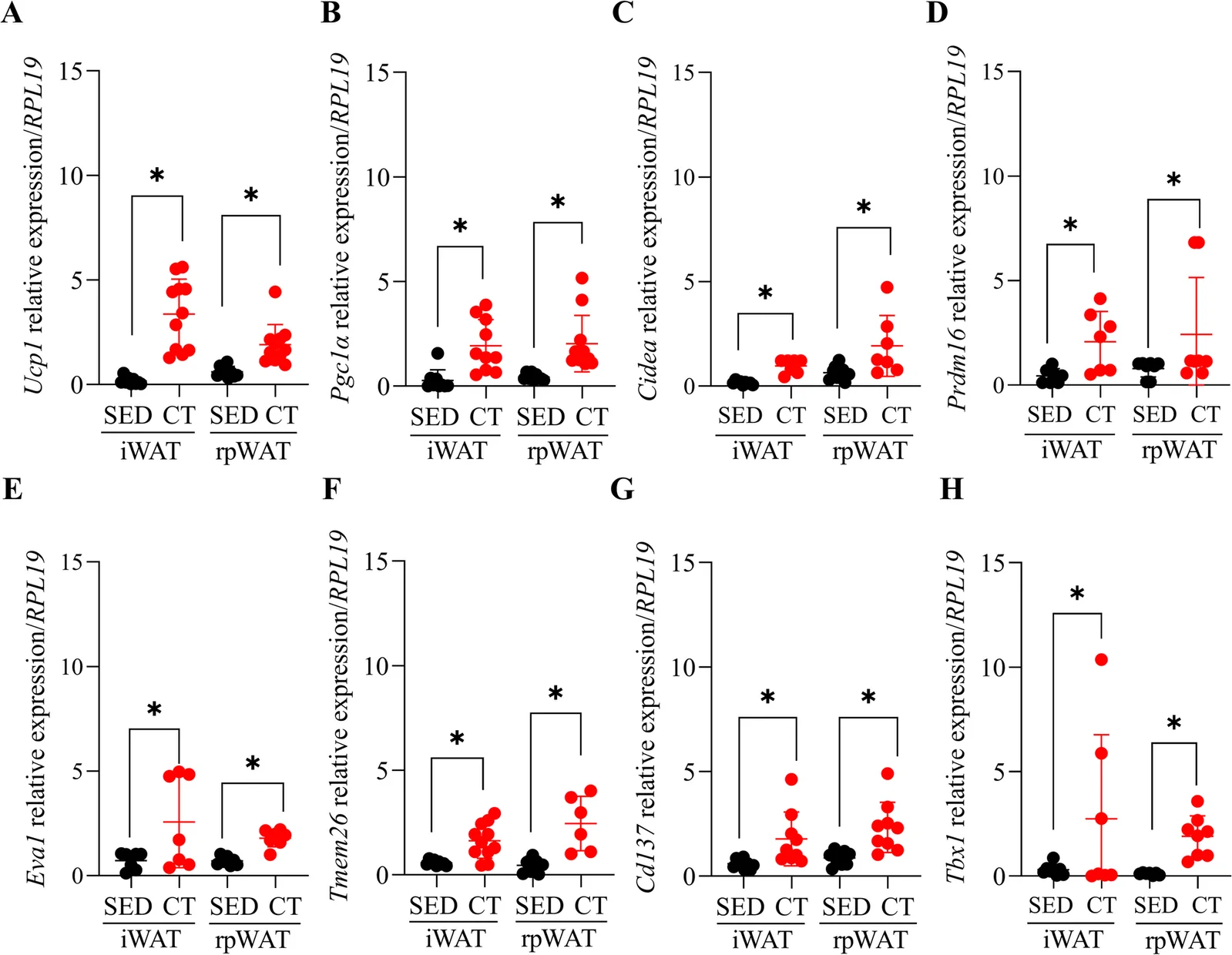
CT increased the expression of selected genes associated with brown and beige adipocyte phenotypes. Gene expression was assessed by qPCR in iWAT and rpWAT. Each data point represents one pooled biological sample generated from two mice. (**A**) Ucp1: SED, n = 9; CT, n = 11. iWAT, q = 0.000165; rpWAT, q = 0.000005. (**B**) Pgc1α: SED, n = 9; CT, n = 10. iWAT, q = 0.0011; rpWAT, q < 0.000001. (**C**) Cidea: SED, n = 9; CT, n = 7. iWAT, q = 0.000549; rpWAT, q = 0.001232. (**D**) Prdm16: iWAT: SED, n = 7; CT, n = 7; q = 0.008779. rpWAT: SED, n = 8; CT, n = 8; q = 0.009927. (**E**) Eva1: SED, n = 7; CT, n = 7. iWAT, q = 0.020144; rpWAT, q = 0.000011. (**F**) Tmem26: iWAT: SED, n = 9; CT, n = 11; q = 0.0011. rpWAT: SED, n = 9; CT, n = 6; q = 0.00047. (**G**) Cd137: SED, n = 9; CT, n = 9. iWAT, q = 0.008779; rpWAT, q = 0.000106. (**H**) Tbx1: iWAT: SED, n = 8; CT, n = 7; q = 0.041811. rpWAT: SED, n = 7; CT, n = 8; q < 0.000001. Data are presented as mean ± SD. Between-group comparisons were performed using Welch’s unpaired t-tests for each gene. Multiple testing across the eight genes was controlled separately within each adipose tissue depot using the two-stage step-up FDR procedure of Benjamini, Krieger, and Yekutieli, with the FDR set at 5% (Q = 0.05). *indicates a significant discovery after FDR correction. Abbreviations: SED, sedentary; CT, combined training; WAT, white adipose tissue; iWAT, inguinal white adipose tissue; rpWAT, retroperitoneal white adipose tissue; qPCR, quantitative polymerase chain reaction; FDR, false discovery rate

After FDR correction, CT significantly increased the expression of all eight brown- and beige-associated genes evaluated, *Ucp1*,* Pgc1α*,* Cidea*,* Prdm16*,* Eva1*,* Tmem26*,* Cd137*, and *Tbx1* in both iWAT and rpWAT. All markers were identified as significant discoveries at Q = 0.05 (Fig. 6).

## Discussion

In the present study, we investigated whether CT could induce a brown-like remodeling of iWAT and rpWAT depots. Our findings demonstrate that CT prescribed within the same week effectively promotes WAT browning as evidenced by enhanced vascularity, UCP1 protein expression, and upregulation of both brown-like and beige gene expression.

The American College of Sports Medicine suggests that the combination of aerobic and resistance exercises promotes additional benefits than just one of these individually [29]. However, evidence for this additive effect is not consistent across the literature and appears to depend on the metabolic status of the population studied. While some studies report additive or synergistic effects of CT, particularly for insulin sensitivity and glycemic control in individuals with obesity, metabolic syndrome, or type 2 diabetes [69], studies conducted in metabolically healthy populations report comparable, rather than superior, effects of CT relative to isolated training modalities [45]. Given that the present study used healthy male Swiss mice, our aim was to determine whether an alternate-day CT protocol could promote browning-related adaptations under physiological conditions.

Nevertheless, CT outcomes may vary according to the contribution of each exercise modality, training intensity and volume, and the temporal organization of aerobic and resistance sessions. Hickson and colleagues were pioneers in demonstrating the effects of concurrent training (same session), i.e., one type of training may lead to an interference effect on the other type [35]. This phenomenon has been associated with metabolic fatigue and reduced performance outcomes, contributing to controversial findings across studies [6, 33, 49]. On the other hand, resistance exercise associated with aerobic exercise can impair strength gain, as well as hypertrophy and muscle power, when compared to resistance training alone [6, 8, 13, 18, 33, 38, 70].

Human studies have demonstrated that CT may reduce fat mass, although the magnitude of this response appears to depend on the population, training volume, intensity, and the organization of aerobic and resistance sessions [10, 19, 21, 27, 39]. In previous CT protocols, aerobic and resistance exercises have been performed within the same session or on the same day [28, 35, 65]. In contrast, the present protocol alternated aerobic and resistance sessions across different days of the same week and alternated the predominant modality between successive weeks. Aerobic intensity and resistance load were also progressively adjusted according to individual performance. Previous studies suggest that the temporal organization of CT may influence the interference response, with same-session or same-day training potentially attenuating some strength- and power-related adaptations compared with protocols in which the modalities are separated [65, 66, 77]. Therefore, separating the modalities across days may provide greater recovery between the distinct exercise stimuli. However, because the present study did not include aerobic-only or resistance-only groups, it cannot establish whether the alternating CT protocol provides greater WAT remodeling or browning than either modality performed alone. Our previous findings showed that aerobic and resistance training performed separately can each promote WAT browning [59], suggesting that the effects observed here may reflect complementary contributions from both stimuli rather than a demonstrated advantage of their combination. Direct comparisons among combined, aerobic-only, and resistance-only protocols are needed to test this possibility.

Within this context, we also evaluated whether the alternating CT protocol was accompanied by changes in body weight. Body weight increased from pre- to post-training in both groups. Although a significant time × training group interaction was detected, no significant differences were observed between SED and CT at either time point. Therefore, this finding should be interpreted cautiously, as it does not support a clear effect of CT on final body weight. The overall increase in body weight likely reflects the normal growth of the mice during the experimental period.

Although exercise increases energy expenditure [31], compensatory increases in food intake are not consistently observed [20]. Exercise may transiently alter appetite regulation and improve satiety signaling, potentially limiting or delaying compensation for the additional energy expenditure. In the present study, food intake decreased significantly from pre- to post-training within the CT group. However, because the time × training group interaction was not significant, the reduction cannot be interpreted as a CT-specific effect relative to the SED group. Nevertheless, because our protocol included forced treadmill running and ladder climbing, a stress-related influence on feeding behavior cannot be excluded. Forced exercise paradigms may activate stress responses, including increased corticosterone, which could alter appetite and feeding behavior [41]. However, stress-related biomarkers and behavioral measures were not assessed in this study; therefore, this explanation remains speculative and should be considered a limitation.

A key cellular response to physical training is angiogenesis, which allows an improvement in capillarization and a better oxygen supply. VEGF and CD31 have been recognized as central regulators of the angiogenic process [14, 25]. In addition, VEGF appears to modulate PGC1α in muscles [15, 46, 62], but also promotes the browning of WAT by increasing the expression of UCP1 in WAT [74]. In our study, CT animals displayed a clear increase in both VEGF and CD31 expression, consistent with changes in angiogenesis-associated markers. These changes, consistent with angiogenic remodeling, may represent an early event in adipose tissue remodeling, facilitating improved oxygen and nutrient delivery within the tissue [2, 51, 55]. Such changes may create a permissive microenvironment that supports the recruitment and activation of beige adipocytes.

While the present study was not designed to directly investigate the underlying mechanisms, it is plausible that both systemic and local factors may contribute to the observed adipose tissue remodeling. Exercise is known to induce the release of circulating mediators such as myokines (e.g., irisin) [1, 17], lactate, and catecholamines, which have been implicated in promoting browning and metabolic adaptations in adipose tissue. In addition, local changes within adipose depots, including enhanced vascularization and oxygen availability, may further support the development of a brown-like phenotype. The decrease in WAT vascularization is well documented in obesity as a result of adipocyte expansion, leading to local hypoxia and low-grade inflammation [34]. Therefore, it is tempting to speculate that CT may help mitigate such phenomena; however, this remains to be confirmed in obese models.

WAT browning may be characterized by at least two distinct adaptations: (1) The presence of smaller multilocular adipocytes, and (2) elevated UCP1 expression [4]. In our work, CT was effective in increasing the expression of UCP1, which was accompanied by the presence of multilocular adipocytes in iWAT and rpWAT. Consistent with these morphological changes, we observed a reduction in adipocyte area; however, no significant differences were detected in iWAT mass between groups. Interestingly, this dissociation may reflect adipose tissue remodeling, potentially involving adipocyte reduction via lipolysis and/or alterations in non-adipocyte components, such as increased stromal or vascular cell content [51, 74]. However, further studies are required to directly assess changes in adipocyte number and the contribution of stromal and vascular components to these observations after CT.

The rise in UCP1 expression increases thermogenic activity, energy expenditure, and fat loss [5]. Interestingly, a previous study conducted in humans submitted to CT did not find any modification in UCP1 expression, but it did find an increase in BAT activity [10]. This apparent discrepancy may be explained by several factors, including species-specific differences in adipose tissue plasticity, variations in tissue sampling (e.g., depot-specific responses), and differences in the sensitivity of detection methods used to assess gene and protein expression [64]. Additionally, emerging evidence suggests that functional activation of thermogenic pathways may occur independently of detectable changes in UCP1 transcript levels under certain conditions [12, 40].

The expression of genes selectively associated with brown and beige adipocyte phenotypes has been described, including *Ucp1*, *Pgc1α*, *Cidea*, *Prdm16*, and *Eva1* for brown adipocytes, and *Tmem26*, *Cd137*, and *Tbx1* for beige adipocytes [24, 67, 78]. In both iWAT and rpWAT, all eight markers, *Ucp1*,* Pgc1α*,* Cidea*,* Prdm16*,* Eva1*,* Tmem26*,* Cd137 and Tbx1* were significantly higher in CT when compared to SED after FDR correction. Although the magnitude of change observed in some genes was modest, these alterations are consistent with the physiological adaptations typically induced by exercise [1, 17]. Importantly, the coordinated regulation of multiple genes related to browning and metabolic activity supports a biologically meaningful remodeling of adipose tissue, rather than isolated effects. Notably, CT significantly increased *Cd137* expression in both WAT depots, a response that was not observed previously when aerobic and resistance exercise were performed separately [59]. We still lack the mechanisms to better understand how the expression of CD137 is associated with the formation of beige adipocytes, since, according to Srivastava [72], CD137 knock-out mice appeared more resistant to WAT browning after cold exposure. Therefore, we suggest further studies using state-of-the-art approaches, including inducible Cre/loxP-mediated systems, CRISPR-Cas9 gene editing, and Cre-dependent iDTR-mediated ablation of specific adipose tissue cell populations, to determine whether targeted manipulation of browning-related genes or cells alters the adipose tissue response to CT.

Abdi and colleagues, in contrast to our experimental design, used the same-day CT protocol in a rat model of type 2 diabetes. Aerobic training was performed in the morning and resistance training in the afternoon, with an 8-hour interval between sessions [1]. The authors found that CT exercise increased fibronectin type III domain-containing 5 (FNDC5) in visceral WAT; however, body composition and phenotypic changes were not observed. Additionally, Pontes and colleagues [79], using rats fed with high-fat diet, adopted CT performed on the same day, but did not observe significant changes in the expression of irisin, PGC1α, PPARγ, or UCP1 in subcutaneous adipose tissue. These findings suggest that, under these conditions, the temporal organization of CT may be one of several factors contributing to the heterogeneous findings. Future studies should directly compare same-day and alternate-day CT protocols while controlling training volume and intensity to determine whether temporal organization influences WAT remodeling.

## Conclusion

**Fig. 7:**
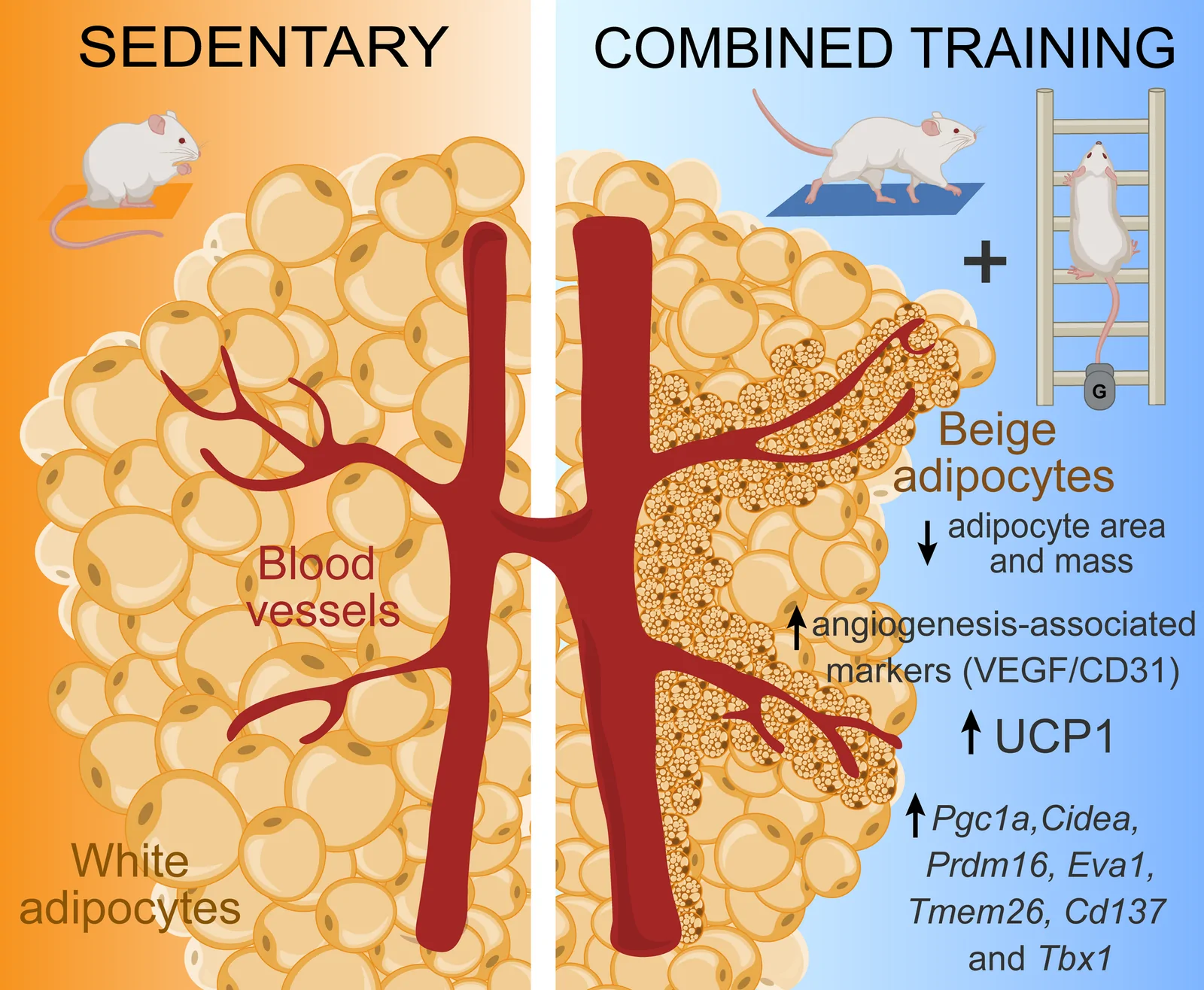
Representative illustration of white adipose tissue (WAT) from sedentary and combined training (CT) mice after 8 weeks of intervention. CT promoted depot-specific changes in WAT mass and reduced adipocyte area, increased angiogenesis-associated markers (VEGF/CD31), presence of multilocular adipocytes expressing UCP1, and upregulation of brown-like and beige adipogenic genes

In summary, eight weeks of CT promoted brown-like remodeling in both iWAT and rpWAT, as demonstrated by changes in angiogenesis-associated markers (VEGF and CD31), UCP1 expression, multilocular adipocytes, and the upregulation of genes associated with brown and beige adipocyte phenotypes. These findings indicate that an alternate-day CT protocol can induce adipose tissue adaptations under physiological conditions (Fig. 7). However, direct comparisons with aerobic-only and resistance-only training are required to determine whether this approach provides additional benefits over isolated exercise modalities.

## Limitations

There are some limitations to this study. The body weight findings should also be interpreted cautiously, as CT did not result in a clear difference in final body weight compared with the SED group. Future studies should incorporate more comprehensive body composition assessments, such as nuclear magnetic resonance or dual-energy X-ray absorptiometry, to better distinguish changes in fat mass, lean mass, and bone mass. Additionally, the RNA pooling strategy used for gene-expression analyses, while necessary to obtain sufficient RNA yield, reduced the effective number of independent biological replicates and precluded assessment of interindividual variability within each experimental group. Future studies should consider individual, non-pooled sampling where feasible to preserve full biological replication. Therefore, future studies should analyze the effects of CT on WAT browning in female mice, to explore the potential sex-specific differences. Additionally, studies using obese mice are needed, as this model may better reproduce the physiological and metabolic environment of obesity, in which adipose tissue dysfunction and systemic inflammation differ from those observed in lean animals. Furthermore, it would be highly valuable to perform RNA sequencing (RNA-seq) and proteomics on different adipose tissue depots after different physical training running in parallel to identify the specific genes and pathways modified by exercise in adipogenesis.

## Data Availability

No datasets were generated or analysed during the current study.
